# The unintended detrimental effects of pursuing a professional vocation: The case of veterinarians

**DOI:** 10.1371/journal.pone.0284583

**Published:** 2023-05-10

**Authors:** Marco A. Palma, Peilu Zhang, Karen Cornell, Matthew Salois, Bridget Bain, Clinton Neill

**Affiliations:** 1 Texas A&M University, College Station, TX, United States of America; 2 Cornell University, Ithaca, NY, United States of America; 3 Veterinary Study Groups, Johns Creek, GA, United States of America; 4 American Society of Composers Authors and Publishers, New York, NY, United States of America; Universitat Jaume I Departament d’Economia, SPAIN

## Abstract

Pursuing one’s life calling can be personally fulfilling and professionally rewarding, but it also requires sacrifice. We provide evidence of a strong vocational drive using veterinary students as a case study and find that they willingly contribute higher monetary donations for helping animals relative to students in other fields. We also find a significant reduction in the cognitive performance of veterinarian students when exposed to an animal-in-need manipulation. The performance of non-veterinary students in the cognitive task is unaffected by the manipulation. Our results highlight the need for programs to address the economic, financial, and mental health well-being of students and professionals to promote sustainable vocational career commitment.

“You owe it to all of us to get on with what you’re good at.” W.H. Auden

## Introduction

When choosing a career, young people are often advised to follow their passion and to focus on making the world a better place. Many career paths, such as law, education, and healthcare, potentially offer higher-than-average compensation and work that is interesting, personally rewarding —and even noble— but they also often fail to meet built-up preconceived expectations [1]. The pursuit of a vocational career may also bring significant mental and physical health burdens due to long working hours, lack of work and life balance to launch and maintain a career, and economic sacrifices to fulfill demanding job expectations [2–4]. Examples include teachers having to use their own resources to buy materials to decorate their classrooms [5], public defenders giving up the opportunity to earn large salaries to pursue a noble cause [6], and nonprofit workers enduring difficult and dangerous conditions to help people in need [7]. These examples highlight how individual’s vocational careers are connected to deeply rooted passions and convictions, sometimes resulting in direct and indirect economic costs. Over time, if not properly managed, high levels of personal and professional stress can lead to career burnout and impaired health [8–13].

Understanding the underlying mechanisms and behavioral drivers that influence an individual’s willingness to accept the costs of such economic and psychological sacrifices is important. For example, the expectations and experience gap in professional or vocational choice can lead to early exit and can also reduce recruitment potential and negatively affect employment opportunities. For instance, fewer individuals are choosing teaching [14] and nursing [15] as a career choice, due in part to all the sacrifice they experience. We evaluate the mental resource process underlying the willingness to take on economic sacrifice associated with following a professional vocation using veterinary medicine as a case study. More specifically, we use a sample of veterinary students to evaluate their monetary donations to an animal charity and their mental focus following an animal-in-distress manipulation. We compare the results from the veterinary students to non-veterinary counterparts living in the same community under similar circumstances. The main hypothesis of our research is that focusing on something individuals deeply care for as it is the case when someone pursues their life calling may sequester significant mental resources to fulfill high demanding jobs, potentially inhibiting the remaining cognitive resource capacity to make other decisions.

The seminal work introduced by Shah et al. (2012) [16] and Mani et al. 2013 [17] documented that focusing on financial scarcity creates preoccupations that inhibit cognitive function. Shah et al.(2012) conducted a series of laboratory experiments to demonstrate that participants with less resources (i.e., scarcity) shift the focus of their attention to more salient and urgent problems, leaving less resources for other tasks [16]. Mani et al. (2013) implement a lab experiment where participants were primed with scarcity of financial resources by asking them how they would confront several unexpected financial expenses. Their results show a substantial reduction in cognitive performance, but only among the “poor”, while the “rich” remain unaffected, presumably because these preoccupations create distractions that predominantly affect low-income individuals, reducing available cognitive resources to perform other activities [17]. Mani et al. (2013) also implemented a field study with sugarcane growers and showed these effects were present among farmers before harvest when they experienced a higher level of scarcity compared to after-the-harvest when they received a boost in their income from selling the sugarcane [17]. There is a growing literature in behavioral economics using scarcity priming to study its effects on financial decisions and cognitive performance. Our research adds to this growing strand of literature, but in a different context for vocational driven focus. We hypothesize that focusing on the attitudinal vocation of a chosen profession captures significant brain resources because of the inherent devotion, lowering the cognitive resources available for other mentally demanding decisions. Our research contributes to this literature, particularly given some recent work that challenges the robustness of the effect size of priming scarcity on cognitive ability. O’Donell et al. (2021) review 20 studies using an empirical audit and review replication approach and while there are differences across studies, they conclude that there are only minimal effects of scarcity priming on cognitive load, but there are likely effects in other financial and consumer decisions [18]. While the behavioral economics literature focuses on scarcity priming of mental resources, an established literature in psychology evaluates the effects of mental effort on resource depletion [19–21].

The *inhibition of mental resources* hypothesis would suggest a causal link between pursuing a vocation and cognitive function. The general concept for the inhibition of mental resources is that people have a pool of available resources that can be allocated to different mental processes [22, 23]. Using resources for one task consumes resources, depleting remaining resources for other tasks. This concept posits that people inhibit the use of mental resources for tasks or processes that are less relevant to conserve the limited resources for more important or immediate endeavors. Dealing with the high demands for job performance and advancement in competitive environments increases the focus for job related tasks, leaving less available resources for other tasks that demand cognitive capacity. In this article, we study veterinary medicine, a noble, well respected, but largely ignored profession. Veterinarians face several serious challenges, including educational debt that is more than triple the average veterinarian’s salary, high levels of career burnout, and far higher rates of suicide compared to the general population [24]. In fact, in a recent study, 42.6% of veterinarians stated they would not recommend the veterinary profession due to excessive levels of educational debt and high workloads [25]. Yet, many veterinary schools in the United States and other countries are experiencing record numbers of applicants seeking admission.

Our study identifies the extent to which students pursuing a veterinary degree 1) are more charitable to animal versus human charities, and 2) quantify any reduction in cognitive performance they experience while fulfilling their professional vocation of helping animals in need compared to students pursuing other disciplines. We tested our research questions in two laboratory experiments. Given the possible sensitive nature of our research question the laboratory provides an ideal environment to test these two questions while having a large degree of control. The first experiment is a dictator game in which participants received a $10 allocation, and they could make a real donation to various charitable causes related to either: 1) the welfare of humans or 2) the welfare of animals. In the second experiment, half of the participants were randomly assigned to a treatment that consisted of watching a short video of an animal-in-distress and were asked to reflect on the best approach to manage the situation, and then were given a cognitive ability test to measure their abstract reasoning and fluid intelligence.

We found evidence of higher donations to animal charities and cognitive function impairment in the students pursuing veterinary medicine when exposed to an animal-in-distress manipulation compared to non-veterinary students and no difference in the donation rates for human charities. Compared to veterinary students, students in other fields made lower donations to animal charities and their cognitive function was unaffected by the animal-in-distress intervention. The findings from this study and key conclusions are potentially not unique to veterinary medicine and can represent many other professions. For example, high levels of commitment, passion, vocation, and burnout rates are common among physicians, pharmacists, nursing, and other professions [26–30]. Perhaps COVID-19 has brought to light this issue among many health professionals [31, 32]. It is important to underscore that the findings from this study are not calling for people to escape their life calling. Rather, the call to action is to increase awareness of the possible pitfalls and challenges people may encounter with respect to their professional vocation even as early as college students. In this way, such informed individuals are then equipped with the knowledge to take the necessary steps to protect themselves against possible negative consequences and support a more sustainable economic, physical, and mental health allowing them to enjoy a longer career and maximize their impact and contributions to their professional field.

## Experimental design

We implement two experiments with two distinct subject populations of veterinary and non- veterinary students. We selected students as our subject population for several reasons. First, college students are a vulnerable population susceptible to considerable stress and mental health challenges that are increasingly receiving attention in the literature [33–36]. Second, students, particularly upperclassmen as in our sample face academic and non-academic related stressors. Third, to facilitate logistical implementation while preserving a high degree of control. The experimental procedures were approved by the Institutional Review Board at Texas A&M University, IRB2020–1278M. Before starting the study, participants provided their written consent. In Experiment 1, a total of 222 students, including 126 non-veterinary students and 96 pre-veterinary/veterinary students participated in two dictator donation tasks. For each donation task, each participant receives a monetary endowment of endowment of $10 (in addition to a $10 participation fee) and is asked to allocate the money (in increments of $1) between him/herself and a charitable organization. We provide five charitable organizations allowing participants to donate to the charity of their choice. In one condition, the five available charities are animal-related, and include: American Society for the Prevention of Cruelty to Animals, American Humane, Best Friends Animal Society, Paws for Ability, and PetSmart Charities. The donations are real, and participants keep the remaining amount from their $10 endowment. The second donation task is identical, except that the five charitable recipients are human-related charities, including: American Red Cross, Feeding America, Scholarship America, Task Force for Global Health, and World Resources Institute. The order of the two donation tasks was randomized to account for ordering effects. The difference in the donation amounts from the animal and human-related charities captures the altruism of veterinary students or the degree of concern for helping animals compared to helping humans. expect veterinary students will have the same degree of concern and contribute similar amounts to human related charities. However, given that veterinary students deeply care for animals, we expect they will contribute to animal charities at higher rates than non-veterinary students.

*Hypothesis 1a*: There is no difference in contributions between veterinary and non-veterinary students for human-related charities.

*Hypothesis 1b*: Veterinary students will contribute a larger amount to the animal-related charities relative to non-veterinary students.

In Experiment 2, the same subjects are randomly assigned to a treatment (64 non-veterinary, 48 veterinary students) or a control (62 non-veterinary, 48 veterinary students) condition. The sample is balanced and there is no significant difference in the demographic characteristics between the control and treatment group for both non-veterinary and veterinary students (See *p*-values in S1 Table). In the treatment, participants watch a short, 20-seconds video, of an animal-in-distress (excerpt from https://www.youtube.com/watch?v=xhLriN3nBdE) and are given five minutes to reflect and write down the best approach to handle the situation. Following the video manipulation, participants perform a Raven’s Progressive Matrices Test [37]. In the control condition, there is no manipulation and participants advance directly to the Raven’s test. We opted for not using a video in the control condition and this was a common feature for veterinary and non-veterinary students. Hence any differences in the treatment and control from the treatment video and the control are identical for each subpopulation. Raven’s Matrices are used to measure fluid intelligence independent of acquired knowledge [38, 39]. For each question, participants are presented with a sequence of shapes and they have 30 seconds to identify the missing element that completes the pattern. In order to ensure participants are attempting to solve the questions, correct answers were incentivized with a monetary reward of a monetary reward of $1 for each correct answer. The inhibition of mental resources hypothesis presented earlier would predict that the animal-in-distress manipulation would only sequester available mental resources for those who deeply care about it and regard it as important. Based on previous work [17] and this theoretical framework, we hypothesize that only veterinary students will engage in inhibitory processes and divert a significant amount of resources to the animal related task compared to non-veterinary students. As a result of this attention shift veterinary students will be preoccupied with the animal-in-distress scenario, leaving less available resources for the raven’s cognitive ability task. Non-veterinary students will not regard the animal-in-distress task with such a high level of importance and will not engage in inhibitory mental resources. As a result non-veterinary students will have the same performance in the raven’s cognitive task in the treatment and control. Our cognitive performance hypotheses are:

*Hypothesis 2a*: The cognitive function of veterinary students will decrease in the animal-in-distress manipulation.

*Hypothesis 2b*: Non-veterinary students will not be affected by the animal-in-distress manipulation.

The experiments were computerized using Qualtrics and had a duration of approximately 30 minutes. Participants earned an average of $25, including a $10 participation fee. The detailed experimental instructions are available in the S1 Table.

## Results

### Experiment 1: Donations to animal and human-related charities

**Fig 1:**
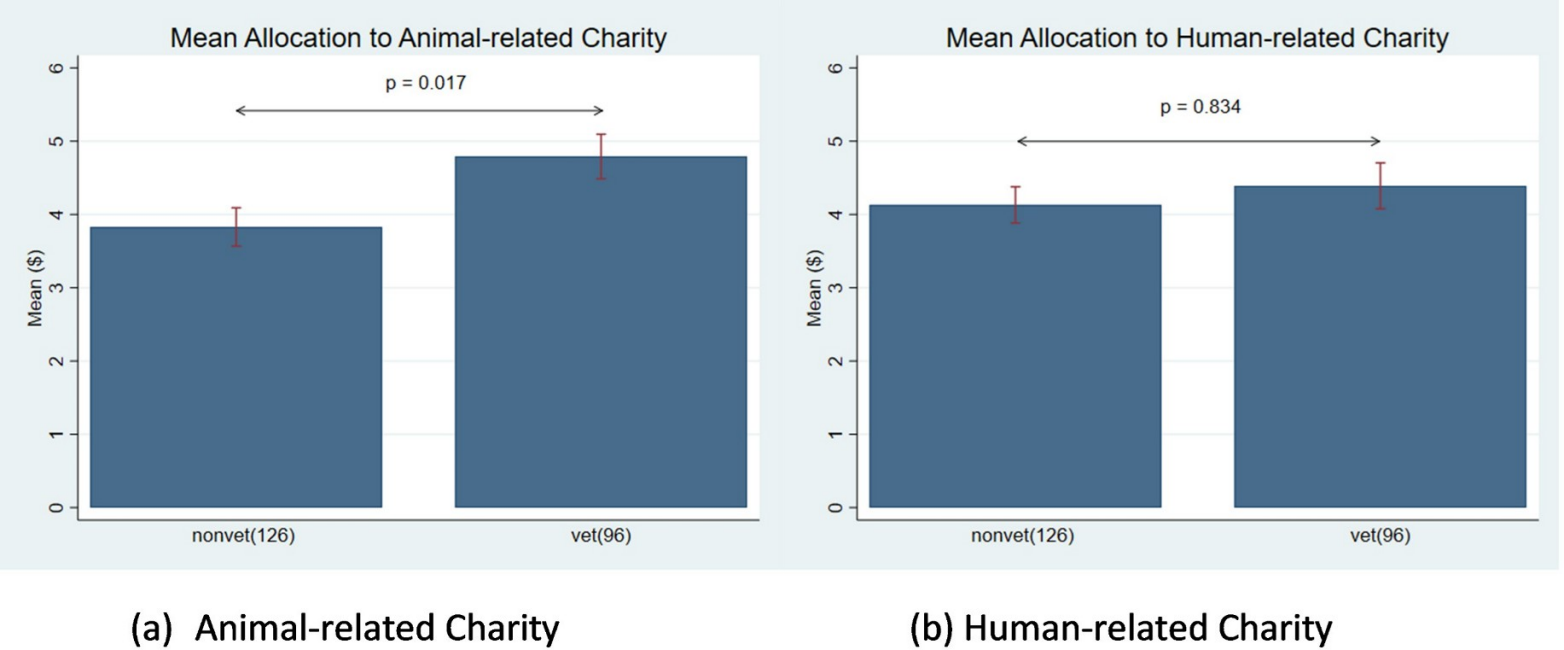
Donations to charities. A: Animal-related charity. B: Human-related charity.

We test Hypotheses 1a and 1b and present the results in Fig 1. For matched data (comparisons across charity types) we used the Wilcoxon signed-rank test and for unmatched data (comparisons across sub-population of veterinary and non-veterinary students) we used the Mann-Whitney test. There is no difference in the contributions for human-related charities between veterinary students, $4.39(*SD* = 3.07), and non-veterinary students, $4.13 (*SD* = 2.77), (Mann-Whitney U-Test, *p* − *value* = 0.834). This result aligns with hypothesis 1a and demonstrates that veterinary and non-veterinary students are equally altruistic towards human related charities. However, veterinary students donate more to the animal-related charities, $4.79(*SD* = 2.97), compared to non-veterinary students, $3.83 (*SD* = 2.93) (Mann-Whitney U-Test, *p* − *value* = 0.017). The difference in donations represent a 25.07% increase in the donation rates by veterinary students. This result provides support for hypothesis 1b and reveals that veterinary students are willing to contribute more to animal-related charities compared to non-veterinary students.

We further find that non-veterinary students keep a larger share of the $10 dictator allocation for themselves compared to their charity donations for both animal and human charities (Wilcoxon signed-rank test, *p* − *value* < 0.001). Veterinary students also keep more money for themselves when asked to allocate the money between themselves and the human-related charity (Wilcoxon signed-rank test, *p* − *value* = 0.019). Interestingly, there is no difference between the contribution of veterinary students to the animal charities and themselves. That is, there is an equal split between veterinary students and the animal charity. This result shows that veterinary students, on average have *egalitarian* views between themselves and the animal charities with the contribution amount not statistically different from the $5 equal split (Wilcoxon signed-rank test, *p* = 0:374). Based on these results, we find quantitative evidence that veterinary students deeply care about animal-related harities, and they are willing to sacrifice their own monetary benefit to help improve the welfare of animals. Our survey results show that on average, veterinary students have 3.2 pets compared to 2.0 pets for non-veterinary students (Mann-Whutney U-Test. *p*−*value* < 0:001), indicating that they self-report to have higher pet care financial costs in the real world, which qualitatively aligns with the results of experiment 1.

Previous literature documents that women tend to be more altruistic than men [40]. Since our sample has a large proportion of women given the composition of veterinary students —with around 80% of female veterinary students in the United States [41]— we present the donation amounts by gender in Table 1. Females in general tend to give larger donations, but there are no differences in the amounts of donation by gender between veterinary and non-veterinary students, although the sample size of male veterinary students is low given the actual composition of the student body population in the veterinary school. Female veterinary students give more to the animal charity compared to the human charity (Wilcoxon signed-rank test, *p* = 0.028). In order to provide robustness to the results, Table 2 presents regression estimates for the donation amounts for each type of charity. The female coefficient is positive and significant for both animal and human related charities, indicating that females send about $1 more in donations than males. This result aligns with previous findings in the literature [40]. The veterinary student coefficient is statistically significant in the animal charity regression with nearly $1 more in donations compared to non-veterinary students. The veterinary student coefficient is not statistically significant in the human charity regression, confirming the results presented above. The donation results are robust to the inclusion of control variables, including gender and other demographic characteristics.

**Table 1:** Donation amounts by gender.

	Female Vet	Female Non-Vet	*p*-value	Male Vet	Male Non-Vet	*p*-value
Animal Charity	$4.91	$4.35	0.226	$4.19	$2.80	0.208
	(2.85)	(2.97)		(3.58)	(2.60)	
Non-animal Charity	$4.44	$4.57	0.453	$4.12	$3.25	0.562
	(2.99)	(2.75)		(3.54)	(2.63)	
*p*-value	0.028	0.110		0.840	0.114	
No. Obs	80	84		16	42	

Note: Standard Deviations in parentheses. The Fall 2021 entry class to the School of Veterinary Medicine at Texas A&M University had a total of 173 students, with 30 males and 143 females, representing 17.3% males and 82.7% females. Our sample has a good representation of veterinary students with around 80% females in the United States. See https://vetmed.tamu.edu/dvm/statistics/ and https://www.vetxinternational.com/male-vs-female-veterinarians-how-the-sexes-compare-in-veterinary-care/

**Table 2:** Regression estimates for donations to the animal and human charities.

	(1) Animal	(2) Human
Vet Student	0.9694**	0.2759
	(0.4348)	(0.4337)
Female	1.2308***	0.9742**
	(0.4516)	(0.4504)
Age	-0.1310	-0.1161
	(0.1019)	(0.1016)
Income		
50*k*−100k	-0.0464	0.3829
	(0.5689)	(0.5674)
Over $100k	-0.0543	0.3299
	(0.5332)	(0.5318)
Household Size	0.1235	0.0388
	(0.1432)	(0.1428)
Race	0.3292	0.2035
	(0.1614)	(0.1609)
Intercept	3.0185	3.8361
	(2.4629)	(2.4566)
No. Obs	222	222

Standard errors in parentheses.

*** *p* < 0.01,

** *p* < 0.05,

* *p* < 0.1

### Experiment 2: Raven’s progressive matrices to measure cognitive performance

**Fig 2:**
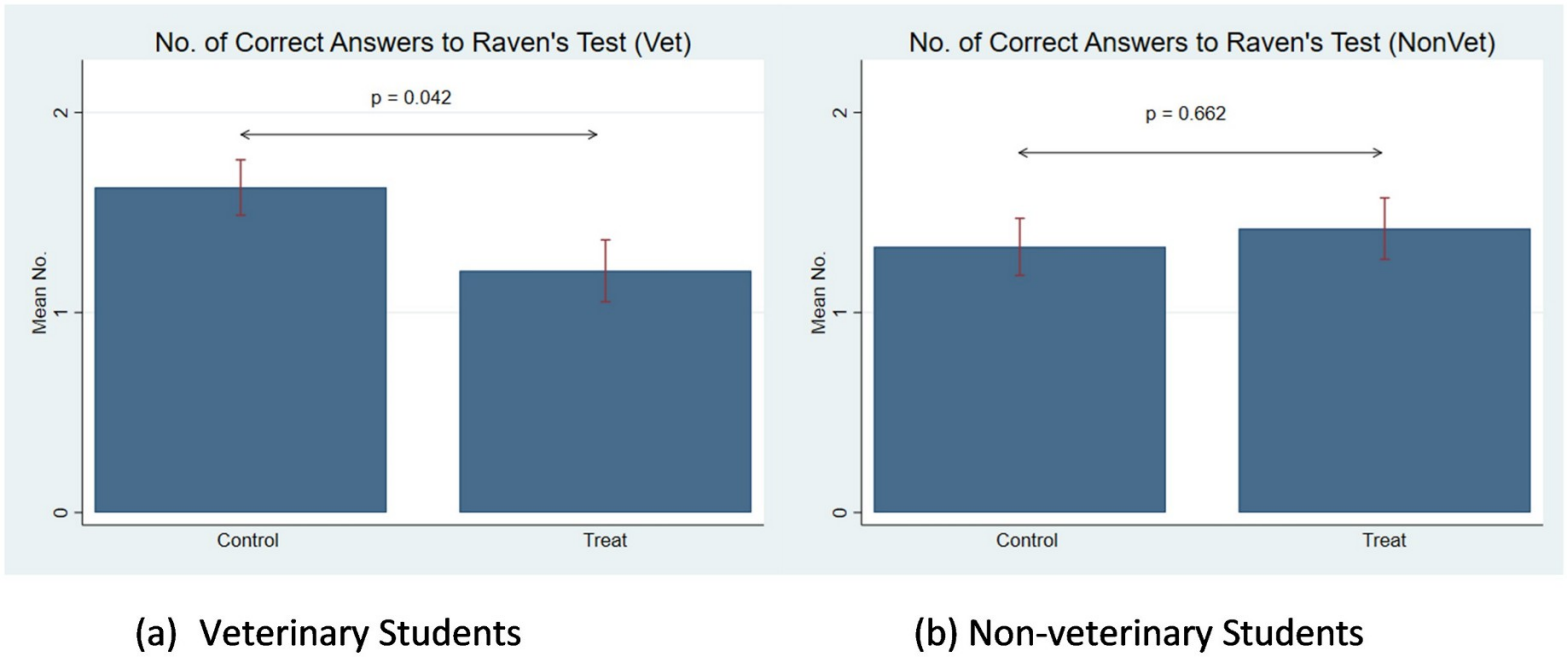
Comparisons of performance in Raven’ metrics between control and treatment. A: Veterinary students. B: Non-veterinary students.

We use a Raven’s Progressive Matrices (RPM) test to measure the cognitive performance of subjects in the animal-in-distress treatment relative to the control condition. Recall that in the treatment we introduce a video and write-up manipulation of how to handle an animal-in-distress situation. There is no manipulation in the control group. Fig 2 shows the results of testing Hypotheses 2a and 2b. The results show that there is no significant effect of the animal-in-distress manipulation on the cognitive function of non-veterinary students (Mann-Whitney U -Test, *p* − *value* = 0.662). However, the animal-in-distress treatment has significant detrimental effects on veterinary students’ cognitive function (Mann-Whitney U -Test, *p* − *value* = 0.042).

The cognitive ability of veterinary students is 23.0% higher in the control group compared to non-veterinary students (Mann-Whitney U -Test, *p* − *value* = 0.079). This result is probably explained by the high selection process in which the highest-performing students are accepted to veterinary medicine school. The cognitive performance of veterinary students in the animal-in-distress manipulation declines 25.8%. The natural advantage veterinary students had in terms of cognitive ability vanished with the animal-in-distress manipulation. In the treatment group, due to the negative effects on veterinary students, the cognitive ability gap between veterinary and non-veterinary students disappeared (Mann-Whitney U -Test, *p* − *value* = 0.412). This result shows that the cognitive performance of veterinary students is impaired when the welfare of animals is at stake. Perhaps more crucially, this finding in such a simple manipulation highlights the potential effects of the scarcity of mental resources to make cognitive demanding decisions among veterinary students in environments where animals are suffering.

We investigate the Raven’s cognitive scores by gender and present the results in Table 3. There is a significant reduction in the cognitive scores of veterinary females in the treatment relative to the control (Mann-Whitney U -Test, *p* − *value* = 0.015). However, there is no significant difference in the cognitive scores of male veterinary students in the treatment compared to the control (Mann-Whitney U -Test, *p* − *value* = 0.569). As expected, there are no significant differences between the treatment and control for female non-veterinary students (Mann-Whitney U -Test, *p* − *value* = 0.387) or male non-veterinary students (Mann-Whitney U -Test, *p* − *value* = 0.969). Notably, the cognitive score for female veterinary students is larger in the control (1.68), compared to non-veterinary students (1.15) (Mann-Whitney U -Test, *p* − *value* = 0.014). This difference disappears in the treatment with cognitive scores of 1.13 and 1.36 for female veterinary and non-veterinary students, respectively (Mann-Whitney U -Test, *p* − *value* = 0.344). In order to investigate the robustness of any potential gender effects driving the results in Table 4, we estimate ordered Probit regressions where the dependent variable is the performance in the Raven’s cognitive test. The regression results provide robustness to the main findings and show a reduction in the Raven’s cognitive scores in the animal-in-distress treatment, but only for veterinary students.

**Table 3:** Raven’s cognitive scores by gender.

	Female Vet	Female Non-Vet	*p-value*	Male Vet	Male Non-Vet	*p-value*
Control	1.68	1.15	0.014	1.29	1.60	0.602
	(0.96)	(1.01)		(0.95)	(1.29	
Treatment	1.13	1.36	0.344	1.56	1.59	0.933
	(1.08)	(1.11)		(1.01)	(1.46)	
*p*-value	0.015	0.387		0.569	0.969	
No. Obs	80	84		16	42	

**Table 4:** Ordered probit regression estimates for Raven’s cognitive scores for veterinary and non-veterinary students.

	(1) Vet Students	(2) Non-Vet Students
Treatment	-0.4176*	0.1268
	(0.2320)	(0.1941)
Female	-0.1344	-0.2692
	(0.2937)	(0.2044)
Age	0.0962**	-0.0135
	(0.0486)	(0.0649)
Income		
50*k*−100k	0.4371	0.1406
	(0.3224)	(0.2913)
Over $100k	0.2522	0.4121
	(0.3159)	(0.2610)
Household Size	0.1010	-0.0638
	(0.0941)	(0.0642)
Race	-0.1181	-0.0691
	(0.1121)	(0.0732)
No. Obs	96	126

Standard errors in parentheses.

*** *p* < 0.01,

** *p* < 0.05,

* *p* < 0.1

The main theoretical framework we presented relies on the mental inhibition hypothesis, in which only veterinary students will devote a significant amount of resources to the animal-in-distress task compared to non-veterinary students and shift their focus of attention and be preoccupied with the animal-in-distress scenario, leaving less available resources for the raven’s cognitive ability task. We hypothesized that non-veterinary students will not engage in the same inhibitory process because they will be able to override it and continue to the Raven’s task with their full mental resource capacity. In order to explore this channel as the potential mechanism behind the results, we analyze the Raven’s cognitive scores for non-veterinary students who donated donated $5 or more to the animal charities (28 subjects), thus demonstrating they deeply care about animal charities, but they may not necessarily engage in the mental resource inhibition process. The results show that the Raven’s scores of non-veterinary students who donated at least $5 are 1:29 in the control and 1:14 in the treatment with the difference not being statistically significant (Mann-Whitney U –Test, p = 0.602). While this result provides support for the attention shift for veterinary students, it doesn’t explain the source of the attention change. A possible explanation is that veterinary students engaged more with the treatment task because they care more about it and as a result had a higher resource depletion causing the reduction in cognitive performance. We analyze the number of words included in the text about how participants would deal with the animal-in-distress scenario as a proxy for effort. On average veterinary and non-veterinary students wrote 79.3 and 73.6 words to describe the way they would handle the animal-in-distress scenario and this difference is not statistically significant (Mann-Whitney U –Test, p = 0.549). Hence, this is not likely to be the case. Another possible explanation is that the animal-in-distress treatment causes more stress or anxiety among veterinary students, thus reducing their cognitive performance in the subsequent task. We did not measure stress or anxiety during the experiment and trying to identify this potential channel is an interesting question for future research. The results provide evidence that the focus of attention shift is not necessarily tied to the effort exerted to comply with the task, but due to preoccupation generated by an attentional focus towards the task among those who deeply care about it.

## Concluding remarks

Overall, the results of hypotheses 1 and 2 show evidence that concentrating on tasks related to pursuing a professional vocation can sequester a significant amount of mental resources leaving less available resources to other non-related tasks. These effects seem to be prevalent among post-secondary students and the literature regarding the mental health and stress of college students seems to be gaining attention [33–36]. The results of our study provide some implications for future work to try to separate the source of stress and mental health burdens arising from academic or job related work and other non-academic or non-job related activities. While a vocational drive is fundamental to the success of individuals, our results highlight the need for programs that aim to help students and professionals cope with academic and job-related tasks, but also with decisions in other dimensions, such as mental burdens, stress, management of time, physical health, monetary and financial management.

The monetary cost and the reduction in cognitive performance effects in our results are quite large. Veterinarian students sacrificed, on average, half of their monetary endowment to donate it to an animal charity foundation. In the animal-in-distress treatment, the cognitive performance of veterinarian students was reduced 25.8% while the cognitive performance of non-veterinarian students remained unaffected. Our results suggest that prospective veterinary students are both determined and arguably, not entirely financially motivated. This altruistic behavior has been labeled by behavioral economists as bounded self-interest [42], a term that proposes that people will often sacrifice their own interests to help others, or in the case of veterinarians, to help animals.

## Supporting information

S1 Appendix Experimental instructions. (PDF)

S1 Table Demographic characteristics. Demographic characteristics of the participants.
(PDF)

S1 Data (ZIP)
