# Supplementary material for: The unintended detrimental effects of pursuing a professional vocation: The case of veterinarians
Source: PLoS One. 2023 May 10;18(5):e0284583. doi: 10.1371/journal.pone.0284583 (PMC10171692; doi:10.1371/journal.pone.0284583)
Supplement: S1 Appendix — (PDF) [file pone.0284583.s001.pdf]

General Instructions

Welcome! Today you will participate in a decision-making study. You will receive $10 as compensation for participating in the study. You will also have the opportunity to receive additional earnings on top of the participation fee based on your decisions and luck. So, please pay attention to the instructions.

Today you will play 4 decision-making tasks. At the end of the session, one of the 4 tasks will be randomly selected for payment.[1] The total amount you earn today will be paid to you in cash and privately at the end of the experiment. After completing the decision-making tasks, we will ask you to fill a questionnaire. Please do not talk during the experiment. All decisions are to be made individually and in private. The information collected will be used solely for research purposes and it is completely anonymous.

Donation Tasks

In this task, you receive an allocation of $10 (in addition to the participation fee). You must decide how to split this money between yourself and a charitable organization. You must allocate any amount of your choice between $0 and $10 (in increments of $1) to a charitable organization, and you keep the remaining amount. You can choose any one of the five charitable organizations to donate by scrolling down the options.

If this task is selected for payment, we will donate the money you allocate to the charitable organization you choose on your behalf. You will keep the remaining amount and it will be paid to you in private at the end of the experiment. Please answer carefully because we will really send the allocated money to the organization you choose. The amount you send will be donated as an anonymous contribution. The organizations will not know your identity.

Animal-related Charitable Organization list:

American Society for the Prevention of Cruelty to Animals.

American Humane.

Best Friends Animal Society.

Paws for Ability.

PetSmart Charities.

Human-related Charitable Organization list:

American Red Cross.

Feeding America.

Scholarship America.

[1]This research article is part of a larger project and the other tasks are designed to evaluate the financial literacy of participants.

Task Force for Global Health.

World Resources Institute.

Questionnaire.

You have completed the decision-making tasks. In this section, you will be asked to complete a survey. You will also have the opportunity to receive additional earnings in some of the survey sections. The information collected will be kept completely anonymous. Please answer the questions carefully and truthfully. Thank you.

Information Treatment

Please watch the following video without clicking anything (the video will end automatically).

Think about what would be the best way to handle the situation in the video. Please write down your answer in the box below. You have up to 5 minutes to answer this question.

Raven's progressive Matrices

In this section, you will be asked to analyze a geometric pattern and identify the missing part to complete the series. First, you will go through an example. Then you will proceed to solve 5 problems. You will receive a compensation of $1 for each correct answer. You have 30 seconds to complete each puzzle.

What is the missing element?

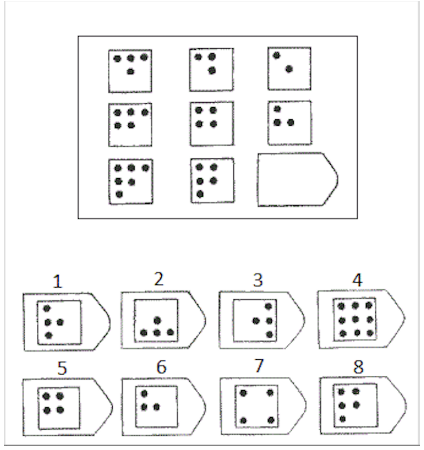

(a) Raven's Matrix 1

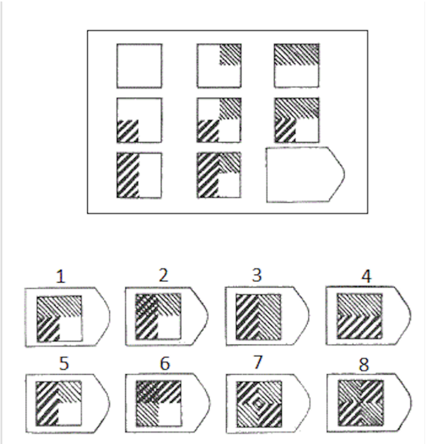

(b) Raven's Matrix 2

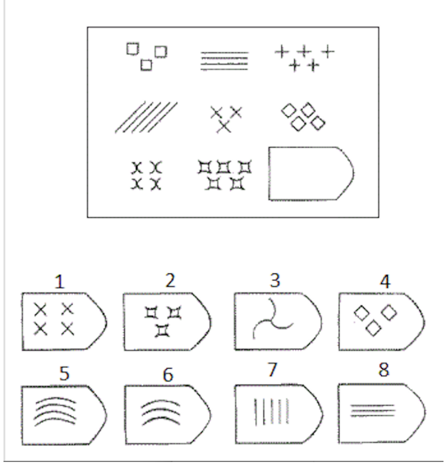

(c) Raven's Matrix 3

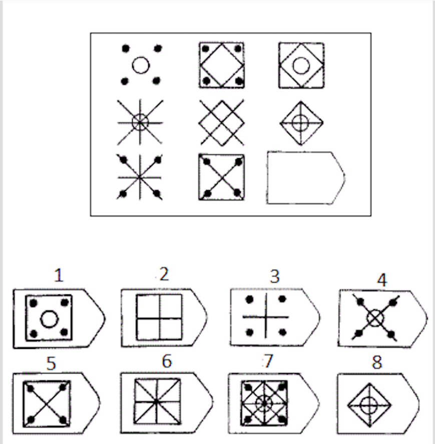

(d) Raven's Matrix 4

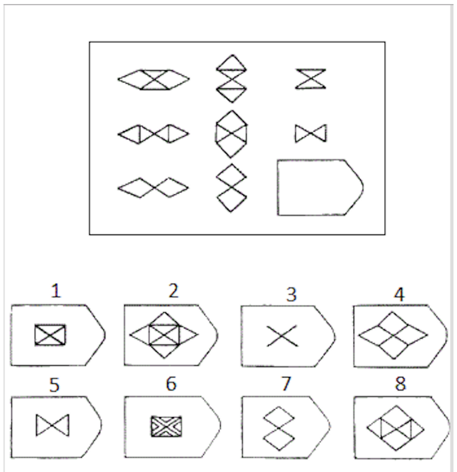

(e) Raven's Matrix 5

Figure S1: Raven's Matrices

Socio-Demographic Survey

Q1. Please enter your age in years.

Q2. Please indicate the HIGHEST level of education you have completed.

A. Some High School or less

B. High School Diploma

C. Some College

D. 2 year/Associates Degree

E. 4 year/Bachelor's Degree

F. Some Graduate School

G.Graduate Degree

Q3. Including yourself, how many people live in your household?

Q4. Please indicate your gender.

A. Male

B. Female

Q5. Please indicate your race.

A. Asian/ Pacific Islander

B. African American

C. Caucasian/ White

D. Native American/ Indigenous

E. Hispanic

F. Other (Please list below)

Q6. Please indicate your family's household yearly income for 2020. (Include all forms of income, including salary, interest and dividend payments, tips, scholarship support, student loans, parental support, and allowance)

A. Less than $50,000

B. $50,000 - $100,000

C. More than $100,000

Q7. Please indicate ALL your current income types (select ALL that apply).

A. Allowance

B. Parental support

C. Salary

D. Scholarship support

E. Student loans

F. Other

Q8. Your college?

A. College of Agriculture and Life Sciences

B. College of Architecture

C. Mays Business School

D. College of Dentistry

E. College of Education & Human Development

F. College of Engineering

G. College of Geosciences

H. Bush School of Government & Public Service

I. School of Innovation

J. School of Law

K. College of Liberal Arts

L. College of Medicine

M. College of Nursing

N. Irma Lerma Rangel College of Pharmacy

O. School of Public Health

P. College of Science

Q. College of Veterinary Medicine & Biomedical Sciences

R. Transition Academic Programs

Q8. Your major?

Q9. How many pets live with you?

0, 1, 2, 3, 4, 5, 6, 7, 8, 9, 10, ¿10

Q10. What the type of pets live with you (select all that apply)? (If you do not have any pet with you, please select n/a.)

A. Birds

B. Cats

C. Dogs

D. Other

E. N/A

Q11. Please indicate the health situation of your pet. (If you do not have any pet, please select any n/a.)

A. All of them are healthy.

B. All of the are sick (needs extra medical care).

C. Some of them are healthy.

D. Some of them are sick.

E. N/A
