# Supplementary material for: The unintended detrimental effects of pursuing a professional vocation: The case of veterinarians
Source: PLoS One. 2023 May 10;18(5):e0284583. doi: 10.1371/journal.pone.0284583 (PMC10171692; doi:10.1371/journal.pone.0284583)
Supplement: S1 Table — Demographic characteristics of the participants. (PDF) [file pone.0284583.s002.pdf]

Supplementary Materials

Demographic Characteristics.

Table S1: Demographic characteristics and balance test of sample participants

	Non-vet (control)	Non-vet (treat)	P-values	Vet (control)	Vet (treat)	P-values
Age (mean)	20	20	0.588	22	22	0.649
Gender	39.1% (F)	72.6% (F)	0.166	85.4% (F)	81.3% (F)	0.584
	60.9% (M)	27.4% (M)		14.6% (M)	18.7% (M)	
Race (White)	60.9%	56.5%	0.656	81.2%	72.9%	0.266
Income(mean)	\$50k-\$100k	\$50k-\$100k	0.565	\$50k-\$100k	\$50k-\$100k	0.352

Notes: The p-values in the last column from two-sided Mann-Whitney U-Tests (Age) and χ^2 tests (Gender, Race, Income).
