# Supplementary material for: The unintended detrimental effects of pursuing a professional vocation: The case of veterinarians
Source: PLoS One. 2023 May 10;18(5):e0284583. doi: 10.1371/journal.pone.0284583 (PMC10171692; doi:10.1371/journal.pone.0284583)
Supplement: S1 Data — (ZIP) [file pone.0284583.s003.zip › final-code-and-data/readme.docx]

Data and Stata Code to replicate the paper, “The Unintended Detrimental Effects of Pursuing a Professional Vocation: The Case of Veterinarians”

This zipfile contains three files.

The vetdata.dta file is the STATA data file. The same dataset is also included as an excel file vetdata.xlsx

The file “veterinarycode” has the basic code to run the statistical tests described in the paper. For questions, please email corresponding author Dr. Marco A. Palma at [mapalma@tamu.edu](mailto:mapalma@tamu.edu)
